# Supplementary material for: Responses and sensitivities of maize phenology to climate change from 1971 to 2020 in Henan Province, China
Source: PLoS One. 2022 Jan 25;17(1):e0262289. doi: 10.1371/journal.pone.0262289 (PMC8789130; doi:10.1371/journal.pone.0262289)
Supplement: Attachment — Submitted filename: Reviewer Recommendation and Comments.docx [file pone.0262289.s003.docx]

**Reviewer Recommendation and Comments**

**for Manuscript Number PONE-D-21-35009**

**Title:** Responses and sensitivities of maize phenology to climate change from 1971 to 2020 in Henan Province, China

Original Submission
 Referee : Majid AghaAlikhan

**ABSTARCT**

- The final sentence of the abstract section should inform the final and practical result of the research. Please rewrite this section.
- **Line 13:** Replace "sunshine": with the "solar radiation". The later one is more professional word to talk about crop yield and its climatic requirements.
- **Line 18**: Correct predicated…. to ……. Predicted.

**KEYWORDS**

- As article keywords: It is recommended to use words that are not used in the title.

**INTRODUCTION**

- **Lines 35:** Replace appropriate seeds to "appropriate genotypes".
- **Line 39:** Replace indexes with " indices"
- **Line 40, 56, 61, 64, 79:** Replace "sunshine": with the "solar radiation".
- **Line 42:** Change**,** Gu and his colleagues… to " Gu et al***.*** It seems the journal guidelines have preferred the number-based citation format, and you are accustomed to reviewing sources, start with citations to the authors' names. It is recommended that you use passive verbs to avoid this inconsistency. For example, instead of "Gu and his colleagues used the climate model," say " the climate model was used ( ). And at the end of the sentence, bring the relevant reference number.
- **Line 43:** same correction as Line 42 is needed for *Gonzales…*.
- **Line 46:** same correction as Line 42 is needed for *Lamptey….*
- **Line 47: Correct Yields ---------🡪 yield**
- **Line 48:** same correction as Line 42 is needed for *Wang's group…..*
- **Line 54:** same correction as Line 42 is needed for *Robin ……*
- **Line 56:** Replace " some scholars with *" a few researcher"*
- **Line 56-61:** no conventional citation: Agroup led by Zhao …. . Same correction as Line 42 is needed for that.
- **Line 62:** same correction as Line 42 is needed for *Yao ……*
- **Line 68:** Delete the repeated word "effect". So rewrite the sentence having the shared word "effect" for all parameters.
- **Line 72:** Instead of pointing directly to the application of this article, which is an unproven claim, say: The practical purpose of this research is to be able to provide a tool for accurately predicting corn yield in the face of climate change.
- **Line 73:** is there difference between maize yield (line 72) and yield of summer maize (line 73)??
- **Line 74:** you applied several models to simulate the weather and meteorological parameters effects on maize yield during a 50 years duration. So these part and their subtitles should be named as **Methids and Materials.**
- **Line 76:** Replave the summer maize with "the crop".
- **Line 79, 82, 121, 123, 124, 126, 128, 129, 130, 133, 136, 137, 138, 140, 162, 163 and 165:** Replace "sunshine": with the "solar radiation".
- **Line 109:** same correction as Line 42 is needed for *Wei and his colleagues ……*
- **Line 122:** Rewrite this simple but important sentence using a better and an academic expression. You severely recommended to emphasizing into the carboxylation pathway of the maize (C4) which indicating its high demand of solar radiation.
- **Line 124-125:** this concept has been repeated in above line. So delete this sentence: Maize need a large amount of sunshine.
- **Line 145:** correct the unit format to : m. s^-1^
- **Line 148:** Replace variety ----------🡪 cultivar
- **Line 150:** Do you mean the wind speed? You mentioned to the wind force!! They are different. If your answer is YES, write the unit for wind speed: m.s^-1^
- **Line 154:** correct the unit format to : m. s^-1^
- **Line 160:** Change elements with: parameters".
- **Line 168:** Replace indexes with " indices"
- **Line 171:** Replace indexes with " indices"
- **Line 178:** Change the paper with the " article"
- **Line 191:** Write the main title for this part of the article **: "Results and Discussion"**
- **Line 200 :** Correct radio -------- > ratio
- **Figurr 1:** correct the unit typesetting as : kg. ha^-1^
- **Line 206:** Replace indexes with " indices"
- **Line 223, 224, 228, 236, 259, 266: 282 and 289 :** Replace "sunshine": with the "solar radiation".
- **Figure 7 and figure 8:** Replace "sunshine": with the "solar radiation".
- **Line 271:** Replace the paper with the "article"

**With best wishes and kind Regards**
