# Supplementary material for: Responses and sensitivities of maize phenology to climate change from 1971 to 2020 in Henan Province, China
Source: PLoS One. 2022 Jan 25;17(1):e0262289. doi: 10.1371/journal.pone.0262289 (PMC8789130; doi:10.1371/journal.pone.0262289)
Supplement: Attachment — Submitted filename: Response to Reviewers.docx [file pone.0262289.s005.docx]

**Journal:** PLOS ONE

**Title:** Responses and sensitivities of maize phenology to climate change from 1971 to 2020 in Henan Province, China

**Manuscript ID:** PONE-D-21-35009

Dear Prof. Vassilis G. Aschonitis,

Thank you very much for your letter that enclosed the reviewers’ comments for our manuscript. We have studied the comments carefully and tried our best to improve the manuscript. We would like to submit the revised manuscript with all revisions marked in blue color. The following are our responses to the reviewers’ comments. Some essential contents have been added in the revised manuscript, however, these changes will not influence the conclusions.

We wish to take this opportunity to thank you and all the reviewers for your consideration and instructive comments. Look forward to hearing from you!

Sincerely,

Ning Zhang

Doctor

Faculty of Forestry, The University of British Columbia,

Vancouver, British Columbia, Canada

**To Reviewer 1**

Reviewer #1: After careful and deep observation of all the authors corrections, for which I am deeply grateful for taking all my comments and recommendations into consideration, I find that the manuscript has now a merit to be published in “PLOS One” in its current form. Authors provided all the reliable sources that were lacking in literature (Introduction), Materials and methods and Discussion parts, adjusted all the sentences that were unclear or badly written in standard English and provided all the supporting data that they were asked for as additional files. I am deeply grateful for this big improvement in the manuscript, that made me completely change my mind in its concern.

Response:

We have studied the comments carefully and tried our best to improve the manuscript. We are very excited that you have changed your mind. All of your comments are very helpful to improve our manuscript. Thank you again.

Best wishes

**To Reviewer 3**

Reviewer #3: I reviewed the authors' responses to my points and comments, as well as their responses to the suggestions of two other respected referees. I hereby confirm that the authors have taken note of all comments made by the reviewers and have made any necessary changes to the text of the revised version of the article. However, I consider it necessary to pay attention to the following two points and make the relevant correction.

Response:

We have studied the comments carefully and tried our best to improve the manuscript. These suggestions are very helpful for us to improve our manuscript. We wish to take this opportunity to thank you for your consideration and instructive comments.

- 1- The author did not understand the meaning of reviewer No. 1, who basically rejected the article, and changed the correct phrase "Climate Change" to the incorrect phrase "weather" in manuscript title, abstract and a few sentences of the body. Do not forget the weather gives temperature, radiation, humidity, etc. in a short period of time, such as 24 a day and 7 day a week. Meanwhile whenever we talk about the status of these parameters in the long run, the term climate should be used. Since this article also uses atmospheric structural data for a period of 50 years, it is appropriate to use the term "climate change" like the original version of the article.
Response:

We agree with your comments on "Climate Change". As you said, we focus on the atmospheric structural data for a period of 50 years. We believe that "Climate Change" is more appropriate. The “weather” has been replaced by "Climate Change" in the revised manuscript.

Thanks very much for your comments.

- 2- Although in the text of the article, the typographical error about the word "ratio" that was written as "radio" have been, the authors forgot to correct this point in the relevant figures as well. In this regard, in Figures 2, 3, 4 and 5 in its vertical axis, the word "radio" should be modified to the word "ratio". There is also a grammatical error in the text of the legend inside the figures. In Figures 2, 3, 4, and 5, which refer to reduced yield in terms of temperature, rainfall, solar radiation, and wind, respectively, in the legend, the word "reduces" must be corrected to "reduced".

Response:

Thanks for your comment. " radio" is a typographical error. We are very sorry that we missed this error when we revised the manuscript for the first time. They have been revised in the updated manuscript. In addition, "reduces" has been corrected to "reduced"
